# Poor access to health services for depression treatment in Brazil

**DOI:** 10.11606/s1518-8787.2023057004654

**Published:** 2023-07-31

**Authors:** Héllyda de Souza Bezerra, Isabelle Ribeiro Barbosa

**Affiliations:** I Universidade Federal do Rio Grande do Norte Programa de Pós-Graduação em Saúde Coletiva Natal RN Brazil Universidade Federal do Rio Grande do Norte. Programa de Pós-Graduação em Saúde Coletiva. Natal, RN, Brazil.; II Universidade Federal do Rio Grande do Norte Faculdade de Ciências da Saúde do Trairi Santa Cruz RN Brazil Universidade Federal do Rio Grande do Norte. Faculdade de Ciências da Saúde do Trairi. Santa Cruz, RN, Brazil

**Keywords:** Depression, Health Services Accessibility, Health Status Disparities, Health Surveys

## Abstract

**OBJECTIVE:**

To analyze the factors associated with poor access to health services for the depression treatment in Brazil.

**METHODS:**

This study used data from the Brazilian National Survey of Health, conducted in the years 2019 and 2020. The sample consisted of 8,332 individuals with a self-reported diagnosis of depression, and poor access to healthcare was identified from the question “what is the main reason for you to not visit the physician/health service regularly for your depression?” From which poor access was identified by the affirmative answer reporting distance of health services or difficulties with transportation; waiting time at the health service; financial difficulties; opening hours of the health service; Not being able to schedule a consultation via health insurance; does not know who to look for or where to go, among others. Sociodemographic aspects and health conditions were analyzed. Bivariate and multivariate analysis was performed using Poisson Regression.

**RESULTS:**

The prevalence of poor access to health services for depression treatment was 14.9% (95%CI: 13.6–16.2), relating to individuals aged 15–29 years (PR = 1.52) and 30-59 years old (PR = 1.22), without education (PR = 1.43), who rate their health as regular/poor/very poor (PR = 1.26), who have some limitation in their usual activities (PR = 2.71), who had the last consultation within 6 months of less than 2 years (PR = 2.63) and for more than 2 years (PR = 2.25) and who do not undergo psychotherapy (PR = 4.28).

**CONCLUSION:**

Poor access to health services for depression treatment was associated with individual factors and health conditions.

## INTRODUCTION

Depression is a mental disorder with various symptoms, of which the main signs and symptoms are sadness, lack of interest, lack of energy, irritability, fatigue, low self-esteem, insomnia or other sleep disorders, cognitive difficulties, and even suicide ideation^1^. Depressive disorders are a serious public health problem worldwide. This is due to the increase in its prevalence and morbidity, leading to repercussions on general health and psychosocial impact^2^. Globally, the number of cases of depression increased 18% between 2005 and 2015, totaling 322 million people diagnosed with depression worldwide. In Brazil, depression affects 11.5 million people (5.8% of the population)^1^, being the fourth leading cause of disability^3^.

In Brazil, the treatment for depression cost less than the socioeconomic impacts generated by the disease, leading to the need for reorganizations of public policies and better health planning for a better direction for the treatment of depression^4^. Access to health services is an important factor of quality and functionality of services. It is configured as a set of aspects that determine the relationship between demand and entry^5^. Despite being a concept that can be interpreted subjectively, access can be comprehensively measured. There is no consensus on the concept of access, several authors measure it differently, such as the use of health services, unmet need, among others^6-8^.

The difficulty in accessing and treating depression is also related to social, economic, and geographic disparities, considering that those who need care the most, such as poor individuals and those who live in regions with limited mental health resources, face greater barriers to receiving adequate care for depression. In Brazil, despite the advancement and expansion of the offer of some mental health services in recent years, difficulties in accessing treatment for depression and other mental disorders still exist, especially in regions with low social and economic conditions^9^.

Given the high prevalence and the increase in morbidity of depression in Brazil, as well as the difficulties in treating it, it is essential to know which factors interfere with the poor access to health care services for depression treatment in the country. Knowing the factors associated with the difficulty in accessing health services will allow a better planning of public mental health policies, as well as a better systematization of the care provided by health services to individuals with depression. Therefore, the objective of this article is to analyze the factors associated with poor access to health services for depression treatment in Brazil.

## METHODS

This study was conducted with data from the 2019 National Survey of Health (*Pesquisa Nacional de Saúde* – PNS), conducted between the years 2019 and 2020, which is a household population survey with the objective of knowing the determinants, conditioning factors, and health needs of the Brazilian population, and thus constitute a representative database of the Brazilian population.

The sampling plan of PNS uses the master sample of the Integrated System of Household Survey (*Sistema Integrado de Pesquisas Domiciliares* – SIPD), which allows greater territorial coverage, and uses a sampling process by conglomerates in three stages, with simple random sampling. The first stage is composed of the Primary Sampling Units (census sectors), the second stage is the selected households, and the third stage is the residents aged 15 years or over selected from each household to answer the survey^10^.

At the end of this process, 94,114 people opted to participate voluntarily in the survey.

The sample for this study consisted of people aged 15 years or over who were selected to answer the survey and who self-reported having a diagnosis for depression. For the selection of the sample, the answer to question Q092 was considered (Has a physician or mental health professional (such as a psychiatrist or psychologist) ever diagnosed you with depression?) Thus, the sample for this study was composed of 8,332 individuals.

In this research, the dependent variable, or primary outcome of the study, was named “poor access to health services for depression treatment.” The outcome expresses the frustration of not having access to depression treatment, within the health care services, due to some accessibility issues.

For the elaboration of the dependent variable, the answer to question Q09502 was considered (“What is the main reason why you do not visit the physician/health service regularly for your depression?”) Individuals who answered at least one of these alternatives were considered: 1) The health service is far away or has difficult with transportation; 2) The waiting time at the health service is too long; 3) Financial difficulties; 4) The opening hours of the health service are incompatible with your work or household activities; 5) You could not schedule a consultation via your health insurance; 6) You do not know who to look for or where to go; and 7) Other reasons.

Among the individual independent variables, sex (male or female), age (15–29 years, 30–59 years, 60 years or older), race/skin color (white, black, indigenous, or Yellow), education level (Higher education, High School, Elementary School, Illiterate), per capita household income (up to 1 minimum wage, 1 to 3 minimum wages, over 3 minimum wages) were considered. The selected individual facilitating factors were: whether the patient is covered by the Family Health Strategy (yes, no, or does not know), place of residence (urban or rural), self-evaluation of health status (very good/good; Regular/Poor/Very poor), whether the patient has been diagnosed with other chronic physical or mental illness, chronic health condition, or long-term illness (yes, no), and whether the patient has limitations in daily activities (such as working, doing household chores, etc.) due to depression (yes, no). Among the variables to characterize access, the following were considered: last time they received medical attention for depression (less than 6 months ago; from 6 months to less than 2 years ago; 2 years ago or more), they undergo psychotherapy (yes, no), use medication for depression (yes, no), and participate in integrative and complementary practice (yes, no). All individual independent variables were collected from the PNS database itself.

The hierarchical model was based on the Framework for the use of health services by people diagnosed with depression in Brazil, based on the model by Andersen^11^.

Since this was a study with complex sampling, the sample weight was used for the analyses and the effect of sample design was incorporated. The prevalence of the outcomes was calculated in relation to the individual variables, with presentation of the respective 95% confidence intervals (95%CI). Subsequently, bivariate Poisson Regression analysis was conducted to estimate the crude prevalence ratio (PR) and the respective 95% confidence interval (95%CI).

The variables that presented p ≤ 0.200 in the bivariate analysis were included in the multivariate Poisson Regression model to estimate the adjusted prevalence ratio (PR). The hierarchical model was adopted, and the variables were entered into the multivariate model according to the increasing order of p-value. Only the variables that were statistically significant (p < 0.05) remained in the final model. All analyses were performed using Stata software, version 13 (Stata Corp., College Station, United States).

The 2019 National Survey of Health project was approved by the National Commission of Ethics in Research (CONEP) of the National Health Council (CNS) of the Ministry of Health, under Opinion no. 3,529,376, dated August 23, 2019. The survey results are in the public domain and are available on the website of the Brazilian Institute of Geography and Statistics. Availability of supplementary data: https://www.ibge.gov.br/estatisticas/sociais/saude/9160-pesquisa-nacional-de-saude.html?edicao=28655&t=resultados

## RESULTS

The prevalence of poor access to health services for depression treatment, that is, individuals with depression who do not regularly go to the physician/health services for treatment due to difficulties in accessing these services, was 14.9% (95%CI 13.6–16.2).

The descriptive analysis of the poor access to health services for the depression treatment showed that the prevalence of this outcome was higher among women, Indigenous and Black individuals, individuals aged from 15 to 29 years, with little to no schooling, individuals with an income of up to 1 minimum wage, who live in rural areas, who evaluate their health as Regular/Poor/Very poor, who have other chronic diseases, who have limitations in their usual activities due to depression, who do not undergo psychotherapy, do not use medication for depression, and who had their last consultation with a physician to treat depression more than 6 months ago (Table 1).

**Table 1:** Prevalence of poor access to health services for the depression treatment according to sociodemographic and health conditions variables in Brazil.

Variables	Descriptive		

	Prevalence (%)	95%CI	P-value
Gender			
Man	14.11	11.86–16.72	0.483
Woman	15.09	13.97–16.28	
Age (year)			
15–29	16.71	13.5–20.5	0.197
30–59 year	14.98	13.65–16.42	
60 or more	13.43	11.89–15.14	
Race or skin color			
White	14.06	12.61–15.64	0.515
Black	15.76	14.31–17.32	
Yellow	14.93	4.508–39.47	
Indigenous	16.05	6.967–32.8	
Education			
Higher Education	12.03	10.01–14.38	< 0.005
Secondary Education	15.79	13.76–18.05	
Primary Education	14.93	13.42–16.59	
Illiterate	22.38	18.44–26.87	
Per capita income			
Up to 1 minimum wage	16.99	15.49–18.6	< 0.005
1–3 minimum wages	14.57	12.94–16.36	
Above 3 minimum wages	9.93	7.514–13.02	
Place of residence			
Rural	15.67	13.76–17.8	0.437
Urban	14.76	13.65–15.96	
Registered at FHS			
Yes	15.57	14.17–17.08	0.119
No	14.23	12.45–16.22	
Does not know	12.48	10.27–15.1	
Self-evaluation of health status			
Very good–Good	12.30	10.85–13.91	< 0.005
Regular–Poor– Very poor	17.29	15.84–18.83	
Has another CNCD			
No	13.72	11.49–16.31	0.333
Yes	15.08	13.91–16.33	
Has activity limitations			
No	10.16	9.045–11.4	< 0.005
Yes	20.68	18.89–22.58	
Last Consultation			
< 6 months	8.60	7.389–9.996	< 0.005
From 6 months to less than 2 years	24.05	21.15–27.2	
2 years or more	16.85	15.22–18.62	
Does psychotherapy			
Yes	3.75	2.622–5.211	< 0.005
No	17.62	16.38–18.94	
Use of medications			
Yes	12.71	11.42–14.12	< 0.005
No	17.30	15.73–19	
Use of ICPs			
Yes	14.58	10.55–19.81	0.904
No	14.88	13.82–16	

95%CI: 95% confidence interval.

CNCD: non-communicable chronic diseases

ICPS: integrative and complementary practices

The analysis of the association between poor access and sociodemographic characteristics and health conditions in the bivariate analysis showed that the outcome was significantly associated with all the surveyed variables. In this analysis, those who evaluate their health as Regular/Poor/Very poor (PR = 1.40; 95%CI 1.20–1.63), who receive up to 1 minimum wage (PR = 1.71 95%CI: 1.28–2.28), and with no schooling (PR = 1.86; 95%CI: 1.42–2.42) stand out (Table 2).

**Table 2:** Crude and adjusted prevalence ratio of poor access to health services for the depression treatment according to sociodemographic and health conditions variables in Brazil.

Variables	Bivariate Analysis			Multivariate Analysis		

	Crude PR	95%CI	p-value	Adjusted PR	95% CI	p-value
Gender						
Man	1		0.485			
Woman	1.06	0.88–1.28				
Age (years)						
15–29	1.24	0.97–1.58	0.076	1.52	1.21–1.91	< 0.005
30–59	1.11	0.95–1.30	0.166	1.22	1.05–1.41	0.008
60 or more	1			1		
Race or skin color						
White	1					
Black	1.12	0.97–1.29	0.121			
Yellow	1.06	0.34–3.26	0.917			
Indigenous	1.14	0.51–2.53	0.744			
Education						
Higher Education	1			1		
Secondary Education	1.31	1.04–1.64	0.019	1.14	0.93–1.40	0.198
Primary Education	1.24	1.00–1.53	0.004	1.04	0.85–1.27	0.697
Illiterate	1.86	1.42–2.42	< 0.005	1.43	1.11–1.83	0.004
Per capita income						
Up to 1 minimum wage	1.71	1.28–2.28	< 0.005			
1–3 minimum wages	1.46	1.09–1.97	0.011			
Above 3 minimum wages	1					
Place of residence						
Rural	1					
Urban	0.94	0.81–1.09	0.437			
Registered at FHS						
Yes	1					
No	0.91	0.77–1.07	0.283			
Does not know	0.80	0.64–0.99	0.042			
Self-evaluation of health status						
Very good - Good	1			1		
Regular - Poor - Very poor	1.40	1.20–1.63	< 0.005	1.26	1.08–1.47	0.003
Has another CNCD						
No	1					
Yes	1.09	0.90–1.33	0.336			
Has activity limitations						
No	1			1		
Yes	2.03	1.75–2.35	< 0.005	2.71	2.29–3.20	< 0.005
Last Consultation						
<6 months	1			1.00		
From 6 months to less than 2 years	2.79	2.20–3.39	< 0.005	2.63	2.15–3.21	< 0.005
2 years or more	1.95	1.63–2.34	< 0.005	2.25	1.82–2.78	< 0.005
Does psychotherapy						
Yes	1			1		
No	4.75	3.34–6.77	< 0.005	4.28	2.99–6.14	< 0.005
Use of medications						
Yes	1					
No	1.36	1.18–1.56	< 0.005			
Use of ICPs						
Yes	1					
No	1.02	0.73–1.41	0.904			

Crude PR: crude prevalence ratio; Adjusted PR: adjusted prevalence ratio; 95%CI: 95% confidence interval.

In the final multivariate analysis model, poor access was associated with being 15–29 years old (PR = 1.52) or being 30–59 years old (PR = 1.22), uneducated (PR = 1.43), those who rated their health as Regular/Poor/Very poor (PR = 1.26), who had some limitation of usual activities due to depression (PR = 2.71), who had their last consultation from 6 months to less than 2 years (PR=2.63) or more than 2 years ago (PR = 2.25), and who do not do psychotherapy (PR = 4.28) (Table 2).

## DISCUSSION

This study showed that the prevalence of poor access to health services for depression treatment was of 14.9%. Surveys conducted in other countries also show a high number of individuals with depression who do not receive treatment due to inadequate access to health services^10,12^, such as one conducted in the United States that showed that 70% of individuals diagnosed with depression did not receive adequate treatment^13^.

A study using data from the 2013 Brazilian National Survey of Health showed that the prevalence of poor access to health services by the Brazilian adult population was 18.1%^14^, a result similar to this study. The study by Dantas et al.^14^ showed that access to health services is still poor for a considerable portion of the Brazilian population, especially the most vulnerable population.

In this study, poor access represents the lack of regular attendance of health services due to accessibility issues. As in the study by Dantas et al.^14^, the precarious access to health services in Brazil shows the frustration of seeking health care, either because they cannot get the care they need or because they were unable to seek the service due to other problems. Several factors may be related to this, such as the characteristics of the health system, individual population factors, geographic characteristics, among others^14^.

Our data point to the existence of inequities in access to depression treatment since some groups of individuals had difficulties in accessing health services, such as individuals aged 15–29 years and 30–59 years, without schooling, who classify their health as regular/poor/very poor, who experience limitation in usual activities due to depression, who had their last consultation between 6 months to less than 2 years or over 2 years, and who do not undergo psychotherapy. A little more than a decade ago, mental health care in primary care was promoted by the mental health policy in Brazil, enabling easier access to users with depression and/or other mental disorders. This policy defined primary care as the main gateway to depression treatment^15,16^.

However, before achieving this access via primary care, Brazilian mental health policy had several other milestones and difficulties. After the psychiatric reform, several services and programs were implemented, such as Psychosocial Care Centers (*Centro de Atenção Psicossocial* – CAPS), the Back to Home Program (*Programa de Volta para Casa*), the Comprehensive Care Program for Users of Alcohol and Other Drugs (*Programa de Atenção Integral a Usuários de àlcool e outras Drogas*), the creation of Psychosocial Care Network (*Rede de Atenção Psicossocial* – RAPS), among others. The implementation of these programs, working together with primary care, brings about a proximity to the users’ life territory, with a continuous bond with the community and longitudinal care^17^.

Another factor that can also influence poor access are issues related to the offer and demand of health services. Worldwide, investments in mental health are limited, resulting in a gap between the need for treatment and its availability^18^.

Despite the advances and expansion of mental health services after the implementation of the RAPS in 2011, Brazil still has significant regional disparities^19^. Regions such as the North and Northeast of Brazil have lower offers of mental health services and primary health care teams when compared with the South and Southeast regions, which have a greater offer of mental health services due to their better economic conditions^20^. Regarding the offer of services and professionals, these regional differences directly impact the access to an early diagnosis and follow-up of individuals with depression.

In the Brazilian public health network, 23.9% of users who access primary care seek care for depression, demonstrating its predominance in mental health care in Brazil^21^. Therefore, access to depression treatment in Brazil should be a priority.

In this study, the factors that remained associated with poor access to depression treatment were: being 15–29 years old or being 30–59 years old, people who are Illiterate, who rate their health as Regular/Poor/Very poor, who have limitations in usual activities due to depression, who had their last consultation from 6 months to less than 2 years or over 2 years ago, and who do not attend psychotherapy.

The prevalence of depression and mental suffering in Brazil, as well as in other countries, is higher among women^22^. This is related to some contributing factors, such as, for example, sociocultural factors, since women are more exposed to the overload of domestic work, domestic violence, and intra-household stressors. With women being divided between multiple roles in society, such as domestic and work activities, they suffer with the high burden of associated factors and symptoms, leading them to require health services^23^. Moreover, women are adapted and taught from an early age of maintaining a health standard related to prevention and self-care^24^, which leads them to seek more health services in most situations.

However, in this study, female sex was not significantly associated in the multivariate analysis. This can be explained by the symptoms of depression in women who are not being monitored and/or treated, because, even though they are culturally more likely to seek health services, depression impacts the ability of individuals to seek self-care, often causing them to not seek treatment due to the symptoms of the disease^25^. In this sense, factors related to mental disorders, such as depression, can create barriers for accessing health services.

As for age, adults aged 30 years or older have a higher prevalence of depression, with a peak between 55 and 74 years^1^, which leads this age group to seek more health services for depression treatment^26^ and may explain the greater frustration when seeking access to health services. This can also be explained by the multiple activities that adults perform in their daily lives, such as work, studies, and children, making it difficult to seek health services^27^.

In turn, adolescents and younger adults have a tendency to not recognize the signs and symptoms of depression^28^, especially adolescents, who most often only seek health services for issues related to the provision of gynecological and obstetric care, as well as actions related to the prevention of pregnancy and sexually transmitted diseases. Adolescents still considers mental illness as a stigma^29^, which leads them to seek health services less, confirming the data of poor access for the age group between 15 and 29 years found in this study.

In our study, race or skin color was not a factor significantly associated with outcome in the final analysis; however, studies demonstrate racial disparities in depression treatment, in which Black and Indigenous people have less access to health care services^30,31^.

The Indigenous population also has less access to health services in Brazil. This may be related to factors such as organizational, geographical, and cultural barriers, including limitations related to the lack of greater communication between ethnic groups and health services^32^. However, even if Black and Indigenous individuals were not associated with the outcome, it must be considered that they still suffer from issues related to poor access in Brazil.

Lower education was associated with higher prevalence of poor access in this study. Education has been considered an important factor associated with better access to adequate treatment for depression. Individuals with more years of schooling are more likely to have treatment for depression since they have more knowledge about the disease and recognize the importance of its treatment. Additionally, individuals with more education have better economic conditions and a greater probability of accessing health services^8^.

Socioeconomic status is also an important factor for access to health services. Although the variable “income” was not associated with the outcome in the multivariate analysis, a study carried out in Brazil shows that individuals who have better access to health services have good economic conditions and private health insurance, which facilitates treatment for chronic diseases. These individuals also report having a complete Higher Education, living in urban areas, and have a good self-reported health status^33^.

Regarding residence, living in rural areas was not associated with the outcome in the multivariate analysis. Notably, most individuals living in rural areas in Brazil have lower economic conditions and less education. Individuals who live in these areas and have mental disorders report that they do not seek health services due to the difficulties of geographical accessibility and to unsatisfactory experiences during the welcoming process^34^. Therefore, we can deduce that living in rural areas is associated with worse socioeconomic conditions in addition to being associated with social exclusion and inequities in health care, especially for individuals with depression.

As for psychotherapy, we included it in the study as an access characterization variable, and not as an outcome variable, although this form of treatment is important for treating depression. This study was based on the behavioral model of Andersen^11^, thus, we chose to include variables that characterize health behavior, such as the search for the use of medications and other therapies.

In this study, individuals who rated their health as regular, bad, or very bad had poor access to depression treatment. Evaluating one’s health as being good is an important indicator of the low impact of depression on the well-being of individuals, which may be linked to adequate treatment^22^. As previously discussed, the individual with untreated depression may lose the ability for self-care.

Depression causes a high burden of disability, leading to limitations in daily activities^1^. Bonadiman et al.^3^ showed in their research that non-adherence to treatment is related to the symptoms that cause limitations in individuals with depression, making them unable to develop their activities, affecting mainly poor women and with low education, confirming the data found in this study.

Lack of psychotherapy may be a consequence of poor access to treatment. Therapy is one of the indispensable resources in the treatment process of an individual with depression. Studies show that adherence to psychotherapy makes the individual understand their health and disease process, leading to a greater demand and/or access to health services for the treatment of depression and other mental disorders^18^.

In addition to the lack of psychotherapy, poor access was also related to non-use of medication and people who had their last consultation more than 6 months ago. The rate of non-use of medication for depression is usually high at the beginning of treatment. Despite this, research shows that the correct use of antidepressants brings clinical improvement to individuals with depression, which makes them adhere to treatment and seek health services for follow-up. Thus, the non-use of medication for depression is an intervening factor in the improvement of the depressive disorder and, consequently, causes individuals with depression not to seek health services, leading to poor access to depression treatment.

Our study has some limitations to be considered. Information bias may interfere in the identification of individuals with self-reported depression; additionally, data referring to the reasons for not seeking services may be subject to the respondent’s memory bias. These situations may have resulted in an underestimation of the prevalence of lack of access. However, the information referred to about access to depression treatment is valid and useful. The results of this survey can serve to compare the Brazilian panorama with that of other countries, in addition to the differential of representativeness at the national level, providing valuable information to support the organization of mental health policies aimed at minimizing the problem herein mentioned.

The results of this study showed that Brazil still face many inequities regarding access to health services for depression treatment; with social, economic, and cultural factors leading Brazilians to have poor access, resulting in a higher prevalence and complications of this mental disorder in Brazil.

This study facilitated the understanding regarding the access to health care services for the depression treatment in Brazil and its relation to the health and living conditions of the population.

## CONCLUSION

The results of this study revealed a prevalence of poor access to depression treatment of 14.9%, a number that can be reduced with the reevaluation of public policies for mental health. Being 15–29 years old or 30–59 years old, not being educated, evaluating their health as Regular/Poor/Very poor, having limitation in usual activities due to depression, having had the last consultation from 6 months to less than 2 years or over 2 years ago, and not doing psychotherapy were the main characteristics that were associated with poor access to depression treatment, in Brazil. Thus, poor access to treatment services for depression, in Brazil, may be related to factors associated with the characteristics of individuals and of the health services.
